# Supplementary material for: “We’ll have to see how it works”: An interview study to understand collaborative practices in interdisciplinary artificial intelligence and healthcare research
Source: PLoS One. 2026 Jul 22;21(7):e0354358. doi: 10.1371/journal.pone.0354358 (PMC13390834; doi:10.1371/journal.pone.0354358)
Supplement: Attachment — Submitted filename: response_to_reviewers.docx [file pone.0354358.s003.docx]

Response to reviewers

**Full reviews:**

**Reviewer #1:**

Dear authors,

Thank you for the opportunity to review the manuscript “Muddying the water: collaborative artificial intelligence practices in healthcare research”, which reports findings from an interview study.

Intro/background
1. Section 2.1 (Collaborations in Healthcare): The content is really about collaborations research and development of health technology—not collaborations in healthcare, which is far too broad to be helpful as it includes clinical care. The point of this section I think is to build on what we know about interdisciplinary collaborations that occur in non-AI health technology, and understand the strengths and challenges of such collaboration.
2. Section 2.2. While it’s interesting to know more about AI and data science work practices, I feel the section should really discuss what limited knowledge we have about collaborative practices within the AI and data science communities. I think the point should focus on insights about challenges, including adapting communication styles (p. 7, line 170). As with 2.1, I believe the point should be to understand interdisciplinary collaborations as they occur within the AI/data science community to inform this manuscript’s own study.
3. Section 2.3. This is an important section, but quite hard to follow. Is the point of this section to show that collaboration in healthcare AI is being increasingly mandated or institutionalised or formalised?

Based on the combined comments from both reviewers, we removed the related work section and condensed it into the introduction. We streamlined it by focusing on the comments made above, especially the second point about section 2.2.

Methods
4. I’m not sure 13 participants is a good number. Please justify this limitation, and include information about the number of staff from the 3 consortia would could have potentially participated? If there’s only 50 combined staff within the consortia, 13 is understandable, but if there’s over a thousand…

Our sample size is based on the size of the research consortia, and Maltreud’s notion of information [1] for sampling in qualitative research. Each consortium had around 20 research staff (from PhD’s to professors). The design of the research points to high information power, and 13 participants is appropriate. First, the aim of the study was narrow focusing specifically on collaboration in interdisciplinary AI research teams. Second, the sample specificity was dense as each of the participants had considerable knowledge of the area of interest. Third, this study is based on established work from CSCW, HCI, and the social sciences. Fourth, the quality of dialogue is strong, with clear communication between researcher and participants. Fifth, analysis is cross-case with participants approached with diverse experiences due to their disciplinary backgrounds. Taken together, this means the study leans towards strong information power, and 13 out of around 60 potential participants being an appropriate sample size. We have added this to the paper on page 6.

[1] Malterud, K., Siersma, V. D., & Guassora, A. D. (2016). Sample Size in Qualitative Interview Studies: Guided by Information Power. *Qualitative health research*, *26*(13), 1753–1760. https://doi.org/10.1177/1049732315617444

5. Please provide information about eligibility criteria for participation.

Any official member of a consortium was eligible, we added a sentence clarifying this in Methods (page 6, line 158).

6. Description of participants: please add information about disciplinary expertise of postdoc researchers and PPIE coordinator. The point of the study is to evaluate multidisciplinary collaboration, so it’s important to see whether there is actual multidisciplinarity among participants. It’s possible that the postdocs are also data scientists.

Where needed, we added discipline in Table 1 (page 7). Most self-identifying postdocs were working from a data science perspective so we included that information. The PPIE coordinators did not have a particular discipline in the strictest definition of a discipline; for these positions it mattered more what kind of role they had rather than the individual background. However, none of them had a data science or clinical background.

Findings
7. Section 4.1 on AI novelty—the first them on “navigating the unknowns” is not as vital as the other two. This part of the section is mainly about working with new technology—but very little to do about what it means for collaboration.

We agree that the first sub-theme is less about collaborations than the other two. However, this sub-theme reflects the setup of the consortia. It contrasts the expectations of participants who may have had experiences with established statistical methods against a new “hyped” technology that had recently exploded in popularity. To address this point we significantly streamlined this sub-theme (page 12, line 219).

8. Section 4.2 “communicating AI”. First, double check the section label because it can be received as communicating “with” AI or “about” AI. I think it’s really just about discussing project updates? Second, the two sub-themes greatly overlap. Data/quotes should make a clearer distinction about the two sub-themes.

We agree there was a significant overlap. We therefore renamed the theme as “Knowledge sharing” and removed the second sub-theme, keeping only one overarching theme (page 16, line 343). The theme includes both project updates as well as discussions that arose from project updates, e.g. a data scientist required clinical input so that their work could continue.

Discussion
9. The claim that “Healthcare data is notoriously difficult to access” is such an insensitive remark. The authors should at least discuss why this is the case, particularly with issues of privacy breaches, misuse of health data, use of data without data subjects’ consent. Lots of examples in the UK!

We agree that the technical challenges in accessing data are directly related to vitally important governance and ethical issues. We have therefore edited the paragraph to provide context to the difficulties of accessing data and why this matters in health data (page 24, line 561).

10. Section 5.2. Communicating AI is such an imprecise label. Better to use something like providing feedback or something similar.

As noted above we renamed and edited the theme.

11. Across the document, the authors keep claiming that the study explores perspectives of multiple academic disciplines – this is overselling the study. If you look at the profile of participants, it looks like it’s mostly data scientists.

Our participant pool reflects the organisation of the consortia and the importance of data scientists in developing AI – they also tended to be the ones working full time on the projects and had a closer sense of what was going in their projects. Additionally, not every data scientist had previous experience with AI, which also brought some interesting viewpoints. Unfortunately we could not capture the perspectives of every discipline involved in each consortia, but we interviewed epidemiologists, statisticians and PPIE coordinators as well. As stated previously, we believe we reached information power with our sample.

**Reviewer #2:**

As a general comment, the introduction reads a little colloquially (e.g. how AI may “muddy the waters”) which may benefit from revision to more technical language (for example, how AI might complicate collaboration). It would also benefit from some discussion about why AI in particular, compared to other tools, is at risk of adding complexity to collaboration.
Some additional exploration in the introduction about why it’s important to understand the way that AI complicates interdisciplinary work would be very helpful

We changed the title of the manuscript and revised the use of the expression.

The review of previous literature is overly long and formatted in an atypical way as an extra section between the introduction and the methods. I would consider shortening it and collapsing it into the introduction to make it more readable.

Some of the introduction is actually an explanation of methods and should probably be moved to the methods (identifying the consortia that participated, etc.)

Similarly, there are things mixed into the methods section (including the mix of participants) that should be reported in the results.

In line with similar comments from R1, we have addressed this by significantly streamlining the related work part into a paragraph as part of the introduction. We also address the other comments by moving the text as suggested by the reviewer (Method section in page 5).

Why were no patients/lay people included in the study (other than PPIE coordinators), even though they are part of the consortia? It seems like that’s an important stakeholder group to capture. I’m glad that PPIE coordinators were included, but the absence of direct patient interviewees is a major limitation. I think the authors need to better justify why they were not included.

It is true that public and patients are an important stakeholder group, but our focus and access on the formal members of consortia. In all the consortia, patients and public were recruited as external participants and most interactions were mediated by the PPIE coordinators. Additionally, at the time of the interviews most of the consortia were still in the process of figuring out the PPIE interactions in relation to AI (which was also still nascent). PPIE coordinators were more closely involved with those developments than the external participants.

A summary of participant demographics (not just role but age, sex etc) should be included to contextualize participant responses.

We did not ask for participants’ age as we did not believe that this was relevant to the questions we raised (as in theory a postdoc could be 24 or 65). Instead, experience with AI and expertise in the fields was more relevant than the age as a number. Additionally, we also did not record gender or sex which again were not relevant to the questions.

Were participants shown the results or given the opportunity to comment after coding was completed?

Participants were not shown results or contacted to comment on findings. As per the information sheet provided, they had the researcher details if they had wanted to contact us about their interviews. No one took up this option.

The authors should consider specifically using and referring to a validated best practice guidance in qualitative research (such as COREG). Many key pieces of information are missing, such as the role and identities of the interviewers. Additionally, some measure of reliability, such as a kappa should be presented.

As required by PLOS ONE, we used the Standards for Reporting Qualitative Research (SRQR) checklist for best practices. Identity and role of interviewers are included in page 8, line 173. We updated the SRQR which is included in the submission. Regarding the measure of reliability, we followed Braun and Clarke’s guide on thematic analysis, which is not predicated on the use of quantitative measures. We additionally followed some of the criteria raised by Cofie et al. (2022) on reliability for qualitative analysis.

1. Braun V, Clarke V. Using thematic analysis in psychology. Qual Res Psychol. 2006 Jan 1;3(2):77–101.

2. Cofie N, Braund H, Dalgarno N. Eight ways to get a grip on intercoder reliability using qualitative-based measures. Can. Med. Ed. J. 2022 Mar. 29;13(2):73-6.

Additionally, was a validated theoretical framework used to guide the interview guide?

We developed the questions following the literature review, using existing questions as examples, our knowledge of data science/epidemiology and designing prompts to guide participants, rather than following an existing framework. We refined the interview protocol via informal conversations with colleagues and a pilot interview with an external participant.

The results are long, with many different quotations used, often one after another. It would be greatly enhanced with some type of figure or at least clear thematic tables to help the reader organize the themes and associated quotations, given the density and breadth of qualitative content.

We reorganised Table 2 (page 10) including a few example quotes to provide more context to the results section.

The study is based exclusively on early-stage UK research consortia, using NHS data. The findings (particularly regarding data governance etc) do not generalize to other national contexts or non-academic health systems.

We agree and discuss these limitations, page 28, line 669.

The discussion could provide better pragmatic “suggestions” as a result of the findings. How do or should these findings impact how consortia working with AI should operate? The manuscript would benefit from more explicit recommendations for improving cross-disciplinary AI collaboration.

Our aim was to unearth “naïve” work practices before the consortia were deep into their AI research. We added a few recommendations in the conclusion section (page 29, line 706). However, a full guideline is beyond our scope and data collection. We believe a new study, comparing end-of-consortia practices and conclusions to early-stage practices, would be in place to provide a complete set of recommendations.
