# Supplementary material for: “We’ll have to see how it works”: An interview study to understand collaborative practices in interdisciplinary artificial intelligence and healthcare research
Source: PLoS One. 2026 Jul 22;21(7):e0354358. doi: 10.1371/journal.pone.0354358 (PMC13390834; doi:10.1371/journal.pone.0354358)
Supplement: Attachment — Submitted filename: minor_revision_comments.docx [file pone.0354358.s004.docx]

Response to Reviewers

--

Review Comments to the Author

Please use the space provided to explain your answers to the questions above. You may also include additional comments for the author, including concerns about dual publication, research ethics, or publication ethics. (Please upload your review as an attachment if it exceeds 20,000 characters)

Reviewer #3: This manuscript presents a qualitative analysis of collaborative AI research in healthcare contexts during formative stages (pre-bedside). The impetus of this work is that newer AI technologies present new opportunities for application in healthcare, and there is little know about challenges and solutions to manage collaboration, which is key to developing translatable AI-powered solutions for patient care and healthcare systems. In the revised manuscript, prior reviewer comments largely addressed. In particular, the streamlined introduction has significantly improved the organization and readability of the paper, as well as the inclusion of information on disciplinary background of participants. The sample size justification is particularly strong. My comments are minor and intended to further strengthen clarity and presentation.

1) At the end of the introduction, the manuscript refers to PPIE Coordinators (second bullet, page 6) before these roles are clearly introduced**. While it is appropriate that detailed information about the consortia was moved to the Methods section, it may be helpful to retain a brief description in the introduction, including a note about the role of the PPIE coordinato**rs. Alternatively, the authors could remove this reference in the introduction and slightly broaden the stated study purpose. Either approach would improve clarity and flow.

*We have updated the second bullet point to reflect that this is staff engaged in PPIE work. This extends further than just PPIE coordinators, as most researchers also engaged in PPIE work and changed practices to work with PPIE contributors. We expand and define the role of PPIE coordinators in the Methods section.*

2) **In the opening of the Findings section (page 16, second sentence),** the statement describing how AI research “impacts those that are not familiar with it” may be overly broad. Consider narrowing this language to better align with the data presented. For **example, the statement could focus on participation in AI research or emphasize participants’ perceptions of AI’s impact, either of which may more accurately reflect the findings.**

*Thanks, we have updated the opening (page 10 in clean version) to reflect the main results more closely and have replaced the line “impacts those that are not familiar with it” with “We discuss how participation in AI research is often novel for participants resulting in different expectations of the capabilities of AI and how this novelty can also amplify the differences between disciplines as stakeholders learn new ways of working.”*

3) **The term National Safe Havens is inconsistently capitalized throughout the manuscript. Please standardize its usage for consistency.**

*Thank you, we have ensured consistency throughout.*

4) While there are different approaches to presenting qualitative findings, one interpretive statement at the end of the first theme (page 24) appears relatively strong for the Findings section. Specifically, the assertion that AI development is a socio technical endeavor influenced by technical factors seems to reflect an a priori assumption stated earlier in the manuscript. I recommend moving this statement to the Discussion, where it could effectively reinforce how the study’s findings align with the conceptual framework guiding the research.

*We have moved the socio-technical sentence to the beginning of the discussion (page 24 in clean version).*

Reviewer #4: Thank you for the opportunity to review the revised manuscript, “‘We’ll have to see how it works’: An interview study to understand collaborative practices in interdisciplinary artificial intelligence and healthcare research.” As a matter of record, I declare no conflicts of interest in reviewing this submission.

I was asked to evaluate the authors’ revised submission given the feedback provided during a prior round of peer review. Accordingly, I have focused primarily on whether the authors have sufficiently addressed the earlier reviewers’ comments, while also noting a few residual issues that remain relevant as they relate to PLOS One’s publication criteria. I found the manuscript substantially improved from its original submission. The authors have made revisions that materially strengthen the conceptual framing, methodological transparency, and presentation of findings.

The authors examine how AI work practices complicate interdisciplinary work in healthcare research, drawing from semi-structured interviews with 13 participants across three UK-based research consortia focused on AI and multimorbidity. Through inductive thematic analysis, they identify three areas of collaborative practice: AI novelty, AI knowledge sharing, and the inclusion of multiple real-world healthcare perspectives. The authors position the study within a sociotechnical theory of AI, where technical choices about models, data, tools, and computational infrastructure are inseparable from the social, institutional, and disciplinary arrangements through which AI development occurs.

This is an interesting and worthwhile setting. Literature around AI in healthcare tends to focus either on the implementation of AI systems in clinical settings or on technical/model performance considerations. As such, the attention given here to the formative stages of AI development prior to deployment is useful. The study’s strongest contribution is that it offers a focused glimpse into how members of AI-healthcare research consortia describe the iterative and often underappreciated work of making AI development understandable across disciplinary boundaries.

The authors have made several meaningful improvements. The revised Introduction is much clearer than the original submission. The original Related Work section has been condensed into a more coherent introductory argument, and the authors now more directly explain why AI specifically complicates interdisciplinary collaboration. The revised manuscript gives greater attention to computational complexity, large dataset requirements, the black-box character of some AI methods, restrictions associated with health data, and the need for various stakeholder perspectives to help interpret or contextualize model development. This is a strong improvement because the paper now better explains why AI is not simply another health technology, but a technology whose development amplifies existing collaborative tensions.

The authors have also improved the methods section. The addition of Malterud et al.’s information power rationale is appropriate for such a qualitative study, and a basic clarification of eligibility criteria is helpful. The authors also added interviewer background, described the coding process with greater clarity, and submitted an SRQR checklist. Likewise, the findings are more accessible now that “Communicating AI” has been renamed and reframed as “AI knowledge sharing.” This resolves an important ambiguity from the earlier manuscript, because the prior label could be read as communication with AI rather than communication about AI-related work. The revised theme is more precise and better linked to the collaborative practices described in the interviews.

The discussion of health data access has also improved considerably. The original wording risked treating data governance, privacy, and access restrictions solely as frustrating barriers. In the revised manuscript, the authors more carefully acknowledge that health records are personal data subject to ethical approvals, anonymization requirements, safe havens, output checking, and disclosure-risk constraints. This revision is important because it preserves the authors’ empirical point, that data access and safe-haven infrastructures shape collaborative AI work, without diminishing the ethical reasons such safeguards exist. Finally, the revised Conclusion now includes more pragmatic suggestions about expectation-setting, AI literacy, support for PPIE coordinators, and resourcing the work of interdisciplinary coordination. These additions are appropriate and help translate the findings into actionable implications.

Notwithstanding these improvements, I believe several minor revisions remain necessary before the manuscript is ready for publication. These are not issues that require new data collection or a major reanalysis. I outline each of these issues below.

(1) Claim Discipline

The first and most important remaining concern is that the manuscript still sometimes overstates the evidentiary basis for claims about patients, patient representatives, and PPIE participants. **The limitation section appropriately acknowledges that no patients from the PPIE groups were interviewed**. This is an important clarification, and I appreciate that the authors have included it**. However, this limitation is not yet carried consistently through the abstract, findings, discussion, and conclusion**. In several places, the manuscript still reads as though direct patient or patient-representative perspectives were captured**, when the direct interview evidence appears to come from consortium members, including PPIE coordinators, rather than from patients or lay contributors themselves**. I have articulated some examples of where this remains an issue in the line-by-line feedback that follows these general comments.

Despite this concern, it is not a fatal issue for the manuscript. Indeed, one of the manuscript’s more interesting findings is that PPIE coordinators appear to take on a translational or knowledge-brokering role by adapting AI-related materials for patient/public audiences and helping researchers understand how such materials may be received. That is an important contribution. **However, the authors should more consistently distinguish between direct interview evidence from PPIE coordinators and indirect accounts of patient/public involvement.** In many places, the manuscript could be strengthened by using language such as “PPIE coordinators’ accounts of patient/public involvement,” “participants’ descriptions of PPIE engagement,” or “reported patient/public contributions,” **rather than phrasing the findings as though patient perspectives were directly elicited.**

A related issue concerns the theme of “multiple real-world healthcare perspectives.” I understand what the authors are trying to accomplish with this theme. Clinicians and patients/public contributors can help contextualize electronic health record data, interpret findings, identify practical relevance, and remind technical teams that data corresponds to real patient experiences. However, in the present study, the patient/public dimension is mediated through PPIE coordinators and consortium participants’ accounts**. The theme can remain, but the evidentiary basis should be stated more carefully**. Otherwise, the paper risks overselling its sample, which was one of the central concerns raised by the initial reviewers.

*Thank you, we have added additional clarity and changed the wording both in the findings (page 10 in the clean version) and discussion (page 24) to highlight that these are reported patient contributions, rather than directly evidenced by patients as interviewees.*

(2) Methodological Transparency

The second remaining issue concerns methodological transparency. The revised methods section is improved, and the SRQR checklist confirms that the authors have attempted to map the manuscript against recognized qualitative reporting standards. Still, several details are clearer in the authors’ response to the initial reviewers than in the manuscript itself. I recommend adding a short paragraph to the methods or limitations section that explicitly addresses four points.

First**, the authors should state whether participant validation or member checking was conducted. The response letter indicates that participants were not shown results or contacted after coding. However, this has not been translated into the revised manuscript.** This does not invalidate the analysis, but it should be disclosed plainly in the manuscript.

*Thank you, we have added a line on page 9 to state that participants were active in the interviews only and were not involved in the coding or discussion of the results.*

Second, the authors should briefly explain why a quantitative inter-coder agreement statistic, such as kappa, was not used. I agree that kappa is not necessarily appropriate for Braun and Clarke-style thematic analysis, particularly when the analysis is inductive and interpretive rather than grounded in a strictly positivist coding ontology. However, **the rationale should appear in the manuscript rather than only in the response letter. The authors can point readers to the procedures they did use to enhance analytic trustworthiness**, such as initial independent coding, harmonization of code vocabulary, codebook refinement, revisiting early transcripts, discussion of coding changes, and joint development of themes.

*We have addressed this comment by including on page 10 a brief rationale related to analytic rigour and the Braun and Clarke methodology, as to why Cohen’s kappa or other statistics were not utilised.*

Third, the authors should clarify what demographic information was and was not collected. The response letter indicates that age, sex, and gender were not collected because the authors viewed role, expertise, and AI experience as more relevant to the study aims. That may be reasonable, particularly in a small and potentially identifiable sample. **However, it should be stated transparently in the manuscript. The authors might also frame this as a balance between contextualizing participants and minimizing deductive disclosure risk in a small sample.**

*We thank the reviewer for highlighting this. On page 9, we have included a sentence on the data collected and why demographic data was not included.*

Fourth, the interview guide is summarized, but not provided. I do not think the absence of the full interview guide prevents publication**, but the manuscript would be strengthened either by including it as supporting information or by giving a somewhat fuller account of how the guide was developed from the literature, prior questions, domain knowledge, informal consultation, and piloting. This would directly respond to the prior reviewer’s concern about whether a validated framework guided the interview protocol.**

*We have added additional details on page 8 ofhow the interview guide was developed and the questions it contained which were based on readings from the literature, as well as informed by the pilot study and feedback from the interdisciplinary research team.*

(3) Sampling-Frame Precision

The third issue is sampling-frame precision. The addition of Malterud et al.’s information power rationale is appropriate and helps justify the sample size. However, the eligible population remains somewhat unclear. The revised manuscript states that each consortium employs around 30 academic and healthcare researchers, while the authors’ response letter refers to around 20 research staff per consortium and frames the sample as 13 participants from approximately 60 potential participants. This may simply reflect different definitions of “research staff,” “formal consortium members,” and broader consortium contributors. However, because the sample-size justification depends partly on the size of the eligible pool, the manuscript should clarify the denominator. If approximately 30 people per consortium includes all academic, clinical, and affiliated contributors, but approximately 20 per consortium refers only to formal research staff practically eligible or reachable for interviews, the authors should state that distinction.

*We have clarified on page 5 each consortia varied in the total number of paid staff, but this sat around 20-30, which does not include the number of PPIE contributors. The number of staff on each consortium also varied considerably given that movement between projects was high, and staff turnover was relatively high which is common for academia where PhD students and post-doc researchers are only present for a set number of years.*

(4) Data Availability

The fourth issue concerns data availability. I did not see a clear manuscript-level data availability statement in the version I reviewed. I recognize that qualitative interview transcripts from a small number of identifiable research consortia may not be appropriate for full public sharing, even if deidentified, because of disclosure risk. That restriction may be entirely justified. However, the manuscript should clearly explain what data can and cannot be shared, and why. At minimum, the authors should specify whether full transcripts are unavailable for confidentiality reasons, whether deidentified excerpts can be made available, and whether the interview guide or codebook can be shared as supporting information.

*As part of the ethical approval sought for this project, this limits our ability to share the data collected. The following data availability statement is part of the structured information in the PLOS One submission, which appears once the manuscript is published:*

*The full interview transcripts are not publicly available. This restriction was a condition of the ethical approval for the study, which specified that access to the raw transcripts would be limited to the researchers directly involved in analysing the data.*

(5) Final Copyediting Considerations

Finally, the manuscript is intelligible and generally written in standard English, but it still needs careful copyediting before publication. The remaining errors do not undermine the study, but they are distracting and should be corrected. PLOS One does not copyedit accepted manuscripts, and I encourage the authors to give the paper a final close review for typographical errors, table formatting, and consistency of terminology.

Specific highlights and points of suggested improvement are listed below:

(Lines 14-28, Abstract) – The abstract is much improved in its framing of the study, but it still overstates patient involvement. The phrase indicating that AI development brings together “data scientists, clinicians, patients and other stakeholders” is broadly true of the consortia, but not of the interview sample. Likewise, the statement that the study shows how “clinicians and patient representatives are involved” should be revised to reflect the actual evidence. A more accurate formulation might refer to how consortium participants, including clinicians and PPIE coordinators, describe the involvement of clinical and patient/public perspectives in early-stage AI development.

(Lines 91-104, Research Question) – The research question remains somewhat awkwardly worded: “how do AI work practices trouble ways of interdisciplinary work in healthcare research?” The meaning is understandable, but the phrasing is not yet polished. Consider revising to something like: “How do AI work practices complicate interdisciplinary collaboration in healthcare research?” or “How do AI work practices shape interdisciplinary collaboration in early-stage healthcare research?”

*We have used “How do AI work practices complicate interdisciplinary collaboration in healthcare research?” moving forward in this paper.*

(**Lines 106-118**, Contributions) – The second contribution statement is much clearer than in the earlier manuscript**, but it should be careful not to overstate patient evidence.** The reference to PPIE coordinators bridging communication between researchers and patients is appropriate. Claims about “ensur[ing] these voices are heard” should be phrased so the reader understands that this conclusion is based on interviews with consortium participants and PPIE coordinators, **not direct interviews with patients/public contributors.**

*We have edited this contribution to ensure it reflects that these findings are the views of PPIE coordinators and people who conduct PPIE in their research, and not direct statements from patients and/or members of the public.*

**(Lines 122-143**, Consortia at a glance) – This section is appropriately located in the methods and gives useful context. **However, the statement that each consortium employs around 30 academic and healthcare researchers should be reconciled with the response-letter statement that each consortium had around 20 research staff**. This matters because it is later used, indirectly, to justify sample size.

*We clarified this section as part of the third point above.*

(**Lines 146-166**, Participants) – The use of Malterud et al.’s information-power framework is appropriate. However, the eligibility sentence requires correction and clarification. **The phrase “reachable my email” appears to be a typographical error. Beyond the typo, the authors should clarify whether “any member of the consortia” includes external PPIE contributors or only formal consortium members**. This clarification is especially important because the absence of direct patient/public interviews is a recurring limitation.

*We clarified that members of the consortia did not involve external PPIE contributors, this is part of our edits throughout the manuscript as well.*

(Table 1, Lines 168-169) – The addition of “Data science” to the two postdoctoral research associates is helpful. **The table formatting could be improved, as the current version is difficult to read.** As a starting point, consider widening the table dimensions to its maximum width.

*We have extended the table width and adjusted some formatting to ensure table readability.*

(**Lines 172-177**, Interviews) – The authors now identify the background of RH and ER, which is helpful. **The development of the interview guide remains somewhat brief. Consider adding one or two sentences describing how the literature, domain knowledge, informal consultation, and pilot interview shaped the final guide.** Alternatively (or additionally), the interview guide could be provided as supporting information.

*We have added more details about this on page 8.*

(**Lines 190-205**, Creation of codebook and analysis) – The coding process is reasonably described. However, “Braun and Clark” should be corrected to “Braun and Clarke.” This section would also benefit from a brief explanation of trustworthiness procedures and the rationale for not reporting kappa. I would not require kappa for this kind of thematic analysis, **but the manuscript should explain why interpretive thematic analysis was better served by iterative discussion, codebook refinement, and joint theme development rather than a quantitative inter-coder reliability statistic.**

*We expanded this point on page 10 with the following comment: “This approach aligns with Braun and Clarke's reflexive thematic analysis, which is grounded in an interpretive paradigm and does not advocate the use of inter-coder reliability statistics as a marker of rigour.”*

**(Lines 208-214**, Findings introduction) – The penultimate sentence of this first paragraph again describes the third theme as involving “clinicians and patients.” Since no patients were interviewed, this should be revised**. “Clinicians and PPIE coordinators’ accounts of patient/public involvement” would be more accurate.**

*This is updated as part of the overall edits regarding the PPIE point.*

**(Lines 342-444**, AI knowledge sharing) – The reframing from “Communicating AI” to “AI knowledge sharing” is a strong revision. This section now more clearly captures how participants adapted tools, formats, and levels of technical detail for different audiences. **Some minor grammatical issues remain, but the analytic structure is clearer.**

(**Lines 484-530,** Relevance to patients) – This is an important part of the findings, but the authors should revise the language to make clear when claims are based on PPIE coordinator accounts rather than direct patient interview evidence. Phrases such as “Patients particularly emphasised” and “Patient collaborators help” should be checked against the available interview data**. If these claims are based on what PPIE coordinators reported, that should be stated.**

*We edited this section to ensure it refers to statements made by PPIE coordinators and not patients.*

(**Lines 624-662**, Additional perspectives in collaborative AI research) – This section contains some of the manuscript’s most interesting discussion, especially the role of PPIE coordinators as translators or knowledge brokers**. However, the language again needs careful calibration.** The study does not directly show how patients influence AI work practices. It shows how consortium participants, including PPIE coordinators, describe patient/public involvement and its mediation through consortium practices.

*This is updated in line with the previous edits as well.*

**(Lines 669-695**, Limitations and future work) – The limitations section appropriately acknowledges the UK/NHS context, early-stage consortia, and the absence of direct patient interviews. This section is helpful. **However, these limitations should be reflected more consistently in the abstract, findings, discussion, and conclusion rather than appearing only toward the end of the paper.**

*We updated earlier sections of the manuscript, including the abstract, to clarify the context and limitations.*

**(Line-level copyediting) – Please correct remaining typographical and grammatical issues, including “reachable my email,” “Braun and Clark,” “Juypter notebook,” and phrases such as “data scientists needed to adjust their adapting communication styles.” Please also check consistency in the use of “multidisciplinary” versus “interdisciplinary,” “PPIE participants” versus “PPIE contributors,” “patient representatives” versus “patients,” and “PPIE coordinators.”**

In summary, the revised manuscript is substantially stronger than the original submission. The authors have clarified the conceptual framing, improved the organization of the manuscript, provided a more defensible sample-size rationale, refined the thematic structure, improved the discussion of health-data governance, and added useful practical recommendations. The remaining revisions are focused and manageable. With these minor revisions, the manuscript would be much better positioned for publication.
