# Supplementary material for: “We’ll have to see how it works”: An interview study to understand collaborative practices in interdisciplinary artificial intelligence and healthcare research
Source: PLoS One. 2026 Jul 22;21(7):e0354358. doi: 10.1371/journal.pone.0354358 (PMC13390834; doi:10.1371/journal.pone.0354358)
Supplement: Peer review history

# Peer review: Decision Letter 0

**DOI:** 10.1371/journal.pone.0354358.r001

**Authors:** Philip A. Cola

31 Oct 2025

PONE-D-25-21991Muddying the water: collaborative artificial intelligence practices in healthcare researchPLOS ONE

Dear Dr. Henkin,

Thank you for submitting your manuscript to PLOS ONE. After careful consideration, we feel that it has merit but does not fully meet PLOS ONE’s publication criteria as it currently stands. Therefore, we invite you to submit a revised version of the manuscript that addresses the points raised during the review process. Both reviewers recognize the importance of your topic and the potential contribution of your findings.  However, major revisions are required to improve clarity, methodological transparency, and conceptual focus of the manuscript.  Below we summary the key required and recommended changes and clarify how to address any issues. Required revisions include reorganization and refocus of the introduction and literature review sections to clearly frame it around interdisciplinary collaboration in AI and data science.  Therefore, also removing or condensing material not directly tied to the research question of this manuscript.  Additionally, we require the strengthening of the methodological information to include eligibility criteria, the total potential participant pool, and demographic characteristics such as role, discipline, age and gender.  It is necessary, to tell us more about who conducted the interviews and describe their relationship to participants, and then add a reference to some standard qualitative reporting framework such as COREQ.  Reviews the presentation of findings to reduce overlapping themes  and clarify how each theme contributes to understanding collaboration.  Add some tables or figures to organize the themes and supporting quotations.  This will help with interpretation.  Finally, please justify participant selection and scope.  Who was not included and why and provide rationale for the sample size in relation to the overall consortia size. We also recommend revising the tone and terminology to avoid colloquial language with more precise or defined academic terminology.  Expand on ethical and governance issues around data access and privacy.  Offer practical recommendation for improving interdisciplinary AI collaboration practices.  Finally, acknowledge that the UK-based setting may limit broader applicability while noting how the the implications will still be generalizable. Please submit your revised manuscript by December 14, 2025. If you will need more time than this to complete your revisions, please reply to this message or contact the journal office at plosone@plos.org. When you're ready to submit your revision, log on to https://www.editorialmanager.com/pone/ and select the 'Submissions Needing Revision' folder to locate your manuscript file.

Please include the following items when submitting your revised manuscript:

- A rebuttal letter that responds to each point raised by the academic editor and reviewer(s). You should upload this letter as a separate file labeled 'Response to Reviewers'.
- A marked-up copy of your manuscript that highlights changes made to the original version. You should upload this as a separate file labeled 'Revised Manuscript with Track Changes'.
- An unmarked version of your revised paper without tracked changes. You should upload this as a separate file labeled 'Manuscript'.

If you would like to make changes to your financial disclosure, please include your updated statement in your cover letter. Guidelines for resubmitting your figure files are available below the reviewer comments at the end of this letter.

If applicable, we recommend that you deposit your laboratory protocols in protocols.io to enhance the reproducibility of your results. Protocols.io assigns your protocol its own identifier (DOI) so that it can be cited independently in the future. For instructions see: https://journals.plos.org/plosone/s/submission-guidelines#loc-laboratory-protocols. Additionally, PLOS ONE offers an option for publishing peer-reviewed Lab Protocol articles, which describe protocols hosted on protocols.io. Read more information on sharing protocols at https://plos.org/protocols?utm_medium=editorial-email&utm_source=authorletters&utm_campaign=protocols.

We look forward to receiving your revised manuscript.

Kind regards,

Philip A. Cola, Ph.D.

Academic Editor

PLOS ONE

Journal Requirements:

When submitting your revision, we need you to address these additional requirements.

1. Please ensure that your manuscript meets PLOS ONE's style requirements, including those for file naming. The PLOS ONE style templates can be found at

https://journals.plos.org/plosone/s/file?id=wjVg/PLOSOne_formatting_sample_main_body.pdf and

https://journals.plos.org/plosone/s/file?id=ba62/PLOSOne_formatting_sample_title_authors_affiliations.pdf

2. We note that the grant information you provided in the ‘Funding Information’ and ‘Financial Disclosure’ sections do not match.

When you resubmit, please ensure that you provide the correct grant numbers for the awards you received for your study in the ‘Funding Information’ section.

3. We note that you have indicated that there are restrictions to data sharing for this study. PLOS only allows data to be available upon request if there are legal or ethical restrictions on sharing data publicly. For more information on unacceptable data access restrictions, please see http://journals.plos.org/plosone/s/data-availability#loc-unacceptable-data-access-restrictions.

Before we proceed with your manuscript, please address the following prompts:

a) If there are ethical or legal restrictions on sharing a de-identified data set, please explain them in detail (e.g., data contain potentially identifying or sensitive patient information, data are owned by a third-party organization, etc.) and who has imposed them (e.g., a Research Ethics Committee or Institutional Review Board, etc.). Please also provide contact information for a data access committee, ethics committee, or other institutional body to which data requests may be sent.

b) If there are no restrictions, please upload the minimal anonymized data set necessary to replicate your study findings to a stable, public repository and provide us with the relevant URLs, DOIs, or accession numbers. For a list of recommended repositories, please see

https://journals.plos.org/plosone/s/recommended-repositories. You also have the option of uploading the data as Supporting Information files, but we would recommend depositing data directly to a data repository if possible.

We will update your Data Availability statement on your behalf to reflect the information you provide.

4. If the reviewer comments include a recommendation to cite specific previously published works, please review and evaluate these publications to determine whether they are relevant and should be cited. There is no requirement to cite these works unless the editor has indicated otherwise.

Additional Editor Comments :

Dear Authors,

Thank you for submitting your manuscript, “Muddying the Water: Collaborative Artificial Intelligence Practices in Healthcare Research.” The reviewers recognize the relevance and potential contribution of your work to understanding collaboration in healthcare AI contexts. However, both reviewers identified substantial issues that must be addressed before the paper can be considered for publication.

Key areas for revision include but are not solely limited to the following:

Clarify the Focus and Framing for the manuscript:

Refocus the background and literature review on interdisciplinary collaboration within AI and data science communities, and how these inform your study context.

Consider integrating the long literature review into the introduction and tightening it (considerable reduction in text would be helpful) around the main research question.

Improve Methodological Transparency:

Provide detailed eligibility criteria, participant demographics (e.g., discipline, age, gender), and the total pool of potential participants.

Clarify the roles and identities of interviewers, and consider referencing a qualitative reporting framework such as COREQ or any other qualitative reporting standard you see fit.

Explain whether participants were given the opportunity to review or comment on findings.

Refine the Presentation of Results:

Simplify and organize the results with clear thematic tables or figures linking themes to quotations.

Review theme definitions for overlap and relevance to the study focus on collaboration (i.e., “Communicating AI”).

Strengthen the Discussion:

Deepen discussion of ethical and contextual issues (e.g., data privacy, access, and governance).

Provide practical implications and recommendations for improving interdisciplinary AI collaborations.

Tone and Style:

Revise colloquial language in the text (i.e., “muddy the waters”) for a more formal, scientific tone.

Ensure claims about multi-disciplinarity are supported by participant data.

In summary, the reviewers see potential value in this work, but significant restructuring and clarification are necessary. If you choose to revise, please prepare a detailed response letter addressing each reviewer comment and explaining how revisions were made.

We look forward to considering a thoroughly revised version of your manuscript. Thank you.

[Note: HTML markup is below. Please do not edit.]

Reviewers' comments:

Reviewer's Responses to Questions

**Comments to the Author**

1. Is the manuscript technically sound, and do the data support the conclusions?

The manuscript must describe a technically sound piece of scientific research with data that supports the conclusions. Experiments must have been conducted rigorously, with appropriate controls, replication, and sample sizes. The conclusions must be drawn appropriately based on the data presented.

Reviewer #1: No

Reviewer #2: Partly

**********

2. Has the statistical analysis been performed appropriately and rigorously?

Reviewer #1: N/A

Reviewer #2: N/A

**********

3. Have the authors made all data underlying the findings in their manuscript fully available?

The PLOS Data policy requires authors to make all data underlying the findings described in their manuscript fully available without restriction, with rare exception (please refer to the Data Availability Statement in the manuscript PDF file). The data should be provided as part of the manuscript or its supporting information, or deposited to a public repository. For example, in addition to summary statistics, the data points behind means, medians and variance measures should be available. If there are restrictions on publicly sharing data—e.g. participant privacy or use of data from a third party—those must be specified.

Reviewer #1: Yes

Reviewer #2: Yes

**********

4. Is the manuscript presented in an intelligible fashion and written in standard English?

PLOS ONE does not copyedit accepted manuscripts, so the language in submitted articles must be clear, correct, and unambiguous. Any typographical or grammatical errors should be corrected at revision, so please note any specific errors here.

Reviewer #1: Yes

Reviewer #2: Yes

**********

5. Review Comments to the Author

Please use the space provided to explain your answers to the questions above. You may also include additional comments for the author, including concerns about dual publication, research ethics, or publication ethics. (Please upload your review as an attachment if it exceeds 20,000 characters)

Reviewer #1: Dear authors,

Thank you for the opportunity to review the manuscript “Muddying the water: collaborative artificial intelligence practices in healthcare research”, which reports findings from an interview study.

Intro/background

1. Section 2.1 (Collaborations in Healthcare): The content is really about collaborations research and development of health technology—not collaborations in healthcare, which is far too broad to be helpful as it includes clinical care. The point of this section I think is to build on what we know about interdisciplinary collaborations that occur in non-AI health technology, and understand the strengths and challenges of such collaboration.

2. Section 2.2. While it’s interesting to know more about AI and data science work practices, I feel the section should really discuss what limited knowledge we have about collaborative practices within the AI and data science communities. I think the point should focus on insights about challenges, including adapting communication styles (p. 7, line 170). As with 2.1, I believe the point should be to understand interdisciplinary collaborations as they occur within the AI/data science community to inform this manuscript’s own study.

3. Section 2.3. This is an important section, but quite hard to follow. Is the point of this section to show that collaboration in healthcare AI is being increasingly mandated or institutionalised or formalised?

Methods

4. I’m not sure 13 participants is a good number. Please justify this limitation, and include information about the number of staff from the 3 consortia would could have potentially participated? If there’s only 50 combined staff within the consortia, 13 is understandable, but if there’s over a thousand…

5. Please provide information about eligibility criteria for participation.

6. Description of participants: please add information about disciplinary expertise of postdoc researchers and PPIE coordinator. The point of the study is to evaluate multidisciplinary collaboration, so it’s important to see whether there is actual multidisciplinarity among participants. It’s possible that the postdocs are also data scientists.

Findings

7. Section 4.1 on AI novelty—the first them on “navigating the unknowns” is not as vital as the other two. This part of the section is mainly about working with new technology—but very little to do about what it means for collaboration.

8. Section 4.2 “communicating AI”. First, double check the section label because it can be received as communicating “with” AI or “about” AI. I think it’s really just about discussing project updates? Second, the two sub-themes greatly overlap. Data/quotes should make a clearer distinction about the two sub-themes.

Discussion

9. The claim that “Healthcare data is notoriously difficult to access” is such an insensitive remark. The authors should at least discuss why this is the case, particularly with issues of privacy breaches, misuse of health data, use of data without data subjects’ consent. Lots of examples in the UK!

10. Section 5.2. Communicating AI is such an imprecise label. Better to use something like providing feedback or something similar.

11. Across the document, the authors keep claiming that the study explores perspectives of multiple academic disciplines – this is overselling the study. If you look at the profile of participants, it looks like it’s mostly data scientists.

Reviewer #2: As a general comment, the introduction reads a little colloquially (e.g. how AI may “muddy the waters”) which may benefit from revision to more technical language (for example, how AI might complicate collaboration). It would also benefit from some discussion about why AI in particular, compared to other tools, is at risk of adding complexity to collaboration.

Some additional exploration in the introduction about why it’s important to understand the way that AI complicates interdisciplinary work would be very helpful

The review of previous literature is overly long and formatted in an atypical way as an extra section between the introduction and the methods. I would consider shortening it and collapsing it into the introduction to make it more readable.

Some of the introduction is actually an explanation of methods and should probably be moved to the methods (identifying the consortia that participated, etc.)

Similarly, there are things mixed into the methods section (including the mix of participants) that should be reported in the results

Why were no patients/lay people included in the study (other than PPIE coordinators), even though they are part of the consortia? It seems like that’s an important stakeholder group to capture. I’m glad that PPIE coordinators were included, but the absence of direct patient interviewees is a major limitation. I think the authors need to better justify why they were not included.

A summary of participant demographics (not just role but age, sex etc) should be included to contextualize participant responses.

Were participants shown the results or given the opportunity to comment after coding was completed?

The authors should consider specifically using and referring to a validated best practice guidance in qualitative research (such as COREG). Many key pieces of information are missing, such as the role and identities of the interviewers. Additionally, some measure of reliability, such as a kappa should be presented.

Additionally, was a validated theoretical framework used to guide the interview guide?

The results are long, with many different quotations used, often one after another. It would be greatly enhanced with some type of figure or at least clear thematic tables to help the reader organize the themes and associated quotations, given the density and breadth of qualitative content.

The study is based exclusively on early-stage UK research consortia, using NHS data. The findings (particularly regarding data governance etc) do not generalize to other national contexts or non-academic health systems.

The discussion could provide better pragmatic “suggestions” as a result of the findings. How do or should these findings impact how consortia working with AI should operate? The manuscript would benefit from more explicit recommendations for improving cross-disciplinary AI collaboration.

**********

6. PLOS authors have the option to publish the peer review history of their article (what does this mean?). If published, this will include your full peer review and any attached files.

If you choose “no”, your identity will remain anonymous but your review may still be made public.

**Do you want your identity to be public for this peer review?**  For information about this choice, including consent withdrawal, please see our Privacy Policy.

Reviewer #1: **Yes:** Yves Saint James Aquino

Reviewer #2: No

**********

[NOTE: If reviewer comments were submitted as an attachment file, they will be attached to this email and accessible via the submission site. Please log into your account, locate the manuscript record, and check for the action link "View Attachments". If this link does not appear, there are no attachment files.]

To ensure your figures meet our technical requirements, please review our figure guidelines: https://journals.plos.org/plosone/s/figures

You may also use PLOS’s free figure tool, NAAS, to help you prepare publication quality figures: https://journals.plos.org/plosone/s/figures#loc-tools-for-figure-preparation.

NAAS will assess whether your figures meet our technical requirements by comparing each figure against our figure specifications.

# Author comment: Author response to Decision Letter 1

**DOI:** 10.1371/journal.pone.0354358.r002

30 Jan 2026

Please see the formatted response in the attached letter..

Response to reviewers

Full reviews:

Reviewer #1:

Dear authors,

Thank you for the opportunity to review the manuscript “Muddying the water: collaborative artificial intelligence practices in healthcare research”, which reports findings from an interview study.

Intro/background

1. Section 2.1 (Collaborations in Healthcare): The content is really about collaborations research and development of health technology—not collaborations in healthcare, which is far too broad to be helpful as it includes clinical care. The point of this section I think is to build on what we know about interdisciplinary collaborations that occur in non-AI health technology, and understand the strengths and challenges of such collaboration.

2. Section 2.2. While it’s interesting to know more about AI and data science work practices, I feel the section should really discuss what limited knowledge we have about collaborative practices within the AI and data science communities. I think the point should focus on insights about challenges, including adapting communication styles (p. 7, line 170). As with 2.1, I believe the point should be to understand interdisciplinary collaborations as they occur within the AI/data science community to inform this manuscript’s own study.

3. Section 2.3. This is an important section, but quite hard to follow. Is the point of this section to show that collaboration in healthcare AI is being increasingly mandated or institutionalised or formalised?

Based on the combined comments from both reviewers, we removed the related work section and condensed it into the introduction. We streamlined it by focusing on the comments made above, especially the second point about section 2.2.

Methods

4. I’m not sure 13 participants is a good number. Please justify this limitation, and include information about the number of staff from the 3 consortia would could have potentially participated? If there’s only 50 combined staff within the consortia, 13 is understandable, but if there’s over a thousand…

Our sample size is based on the size of the research consortia, and Maltreud’s notion of information [1] for sampling in qualitative research. Each consortium had around 20 research staff (from PhD’s to professors). The design of the research points to high information power, and 13 participants is appropriate. First, the aim of the study was narrow focusing specifically on collaboration in interdisciplinary AI research teams. Second, the sample specificity was dense as each of the participants had considerable knowledge of the area of interest. Third, this study is based on established work from CSCW, HCI, and the social sciences. Fourth, the quality of dialogue is strong, with clear communication between researcher and participants. Fifth, analysis is cross-case with participants approached with diverse experiences due to their disciplinary backgrounds. Taken together, this means the study leans towards strong information power, and 13 out of around 60 potential participants being an appropriate sample size. We have added this to the paper on page 6.

[1] Malterud, K., Siersma, V. D., & Guassora, A. D. (2016). Sample Size in Qualitative Interview Studies: Guided by Information Power. Qualitative health research, 26(13), 1753–1760. https://doi.org/10.1177/1049732315617444

5. Please provide information about eligibility criteria for participation.

Any official member of a consortium was eligible, we added a sentence clarifying this in Methods (page 6, line 158).

6. Description of participants: please add information about disciplinary expertise of postdoc researchers and PPIE coordinator. The point of the study is to evaluate multidisciplinary collaboration, so it’s important to see whether there is actual multidisciplinarity among participants. It’s possible that the postdocs are also data scientists.

Where needed, we added discipline in Table 1 (page 7). Most self-identifying postdocs were working from a data science perspective so we included that information. The PPIE coordinators did not have a particular discipline in the strictest definition of a discipline; for these positions it mattered more what kind of role they had rather than the individual background. However, none of them had a data science or clinical background.

Findings

7. Section 4.1 on AI novelty—the first them on “navigating the unknowns” is not as vital as the other two. This part of the section is mainly about working with new technology—but very little to do about what it means for collaboration.

We agree that the first sub-theme is less about collaborations than the other two. However, this sub-theme reflects the setup of the consortia. It contrasts the expectations of participants who may have had experiences with established statistical methods against a new “hyped” technology that had recently exploded in popularity. To address this point we significantly streamlined this sub-theme (page 12, line 219).

8. Section 4.2 “communicating AI”. First, double check the section label because it can be received as communicating “with” AI or “about” AI. I think it’s really just about discussing project updates? Second, the two sub-themes greatly overlap. Data/quotes should make a clearer distinction about the two sub-themes.

We agree there was a significant overlap. We therefore renamed the theme as “Knowledge sharing” and removed the second sub-theme, keeping only one overarching theme (page 16, line 343). The theme includes both project updates as well as discussions that arose from project updates, e.g. a data scientist required clinical input so that their work could continue.

Discussion

9. The claim that “Healthcare data is notoriously difficult to access” is such an insensitive remark. The authors should at least discuss why this is the case, particularly with issues of privacy breaches, misuse of health data, use of data without data subjects’ consent. Lots of examples in the UK!

We agree that the technical challenges in accessing data are directly related to vitally important governance and ethical issues. We have therefore edited the paragraph to provide context to the difficulties of accessing data and why this matters in health data (page 24, line 561).

10. Section 5.2. Communicating AI is such an imprecise label. Better to use something like providing feedback or something similar.

As noted above we renamed and edited the theme.

11. Across the document, the authors keep claiming that the study explores perspectives of multiple academic disciplines – this is overselling the study. If you look at the profile of participants, it looks like it’s mostly data scientists.

Our participant pool reflects the organisation of the consortia and the importance of data scientists in developing AI – they also tended to be the ones working full time on the projects and had a closer sense of what was going in their projects. Additionally, not every data scientist had previous experience with AI, which also brought some interesting viewpoints. Unfortunately we could not capture the perspectives of every discipline involved in each consortia, but we interviewed epidemiologists, statisticians and PPIE coordinators as well. As stated previously, we believe we reached information power with our sample.

Reviewer #2:

As a general comment, the introduction reads a little colloquially (e.g. how AI may “muddy the waters”) which may benefit from revision to more technical language (for example, how AI might complicate collaboration). It would also benefit from some discussion about why AI in particular, compared to other tools, is at risk of adding complexity to collaboration.

Some additional exploration in the introduction about why it’s important to understand the way that AI complicates interdisciplinary work would be very helpful

We changed the title of the manuscript and revised the use of the expression.

The review of previous literature is overly long and formatted in an atypical way as an extra section between the introduction and the methods. I would consider shortening it and collapsing it into the introduction to make it more readable.

Some of the introduction is actually an explanation of methods and should probably be moved to the methods (identifying the consortia that participated, etc.)

Similarly, there are things mixed into the methods section (including the mix of participants) that should be reported in the results.

In line with similar comments from R1, we have addressed this by significantly streamlining the related work part into a paragraph as part of the introduction. We also address the other comments by moving the text as suggested by the reviewer (Method section in page 5).

Why were no patients/lay people included in the study (other than PPIE coordinators), even though they are part of the consortia? It seems like that’s an important stakeholder group to capture. I’m glad that PPIE coordinators were included, but the absence of direct patient interviewees is a major limitation. I think the authors need to better justify why they were not included.

It is true that public and patients are an important stakeholder group, but our focus and access on the formal members of consortia. In all the consortia, patients and public were recruited as external participants and most interactions were mediated by the PPIE coordinators. Additionally, at the time of the interviews most of the consortia were still in the process of figuring out the PPIE interactions in relation to AI (which was also still nascent). PPIE coordinators were more closely involved with those developments than the external participants.

A summary of participant demographics (not just role but age, sex etc) should be included to contextualize participant responses.

We did not ask for participants’ age as we did not believe that this was relevant to the questions we raised (as in theory a postdoc could be 24 or 65). Instead, experience with AI and expertise in the fields was more relevant than the age as a number. Additionally, we also did not record gender or sex which again were not relevant to the questions.

Were participants shown the results or given the opportunity to comment after coding was completed?

Participants were not shown results or contacted to comment on findings. As per the information sheet provided, they had the researcher details if they had wanted to contact us about their interviews. No one took up this option.

The authors should consider specifically using and referring to a validated best practice guidance in qualitative research (such as COREG). Many key pieces of information are missing, such as the role and identities of the interviewers. Additionally, some measure of reliability, such as a kappa should be presented.

As required by PLOS ONE, we used the Standards for Reporting Qualitative Research (SRQR) checklist for best practices. Identity and role of interviewers are included in page 8, line 173. We updated the SRQR which is included in the submission. Regarding the measure of reliability, we followed Braun and Clarke’s guide on thematic analysis, which is not predicated on the use of quantitative measures. We additionally followed some of the criteria raised by Cofie et al. (2022) on reliability for qualitative analysis.

1. Braun V, Clarke V. Using thematic analysis in psychology. Qual Res Psychol. 2006 Jan 1;3(2):77–101.

2. Cofie N, Braund H, Dalgarno N. Eight ways to get a grip on intercoder reliability using qualitative-based measures. Can. Med. Ed. J. 2022 Mar. 29;13(2):73-6.

Additionally, was a validated theoretical framework used to guide the interview guide?

We developed the questions following the literature review, using existing questions as examples, our knowledge of data science/epidemiology and designing prompts to guide participants, rather than following an existing framework. We refined the interview protocol via informal conversations with colleagues and a pilot interview with an external participant.

The results are long, with many different quotations used, often one after another. It would be greatly enhanced with some type of figure or at least clear thematic tables to help the reader organize the themes and associated quotations, given the density and breadth of qualitative content.

We reorganised Table 2 (page 10) including a few example quotes to provide more context to the results section.

The study is based exclusively on early-stage UK research consortia, using NHS data. The findings (particularly regarding data governance etc) do not generalize to other national contexts or non-academic health systems.

We agree and discuss these limitations, page 28, line 669.

The discussion could provide better pragmatic “suggestions” as a result of the findings. How do or should these findings impact how consortia working with AI should operate? The manuscript would benefit from more explicit recommendations for improving cross-disciplinary AI collaboration.

Our aim was to unearth “naïve” work practices before the consortia were deep into their AI research. We added a few recommendations in the conclusion section (page 29, line 706). However, a full guideline is beyond our scope and data collection. We believe a new study, comparing end-of-consortia practices and conclusions to early-stage practices, would be in place to provide a complete set of recommendations.

Attachment Submitted filename: response_to_reviewers.docx

# Peer review: Decision Letter 1

**DOI:** 10.1371/journal.pone.0354358.r003

**Authors:** Philip A. Cola

21 May 2026

PONE-D-25-21991R1“We’ll have to see how it works”: An interview study to understand collaborative practices in interdisciplinary artificial intelligence and healthcare researchPLOS One

Dear Dr.  Henkin,

Thank you for submitting your manuscript to PLOS ONE. After careful consideration, we feel that it has merit but does not fully meet PLOS ONE’s publication criteria as it currently stands. Therefore, we invite you to submit a revised version of the manuscript that addresses the points raised during the review process.

Please submit your revised manuscript by Jul 05 2026 11:59PM. If you will need more time than this to complete your revisions, please reply to this message or contact the journal office at plosone@plos.org. When you're ready to submit your revision, log on to https://www.editorialmanager.com/pone/ and select the 'Submissions Needing Revision' folder to locate your manuscript file.

Please include the following items when submitting your revised manuscript:

- A letter that responds to each point raised by the academic editor and reviewer(s). You should upload this letter as a separate file labeled 'Response to Reviewers'.
- A marked-up copy of your manuscript that highlights changes made to the original version. You should upload this as a separate file labeled 'Revised Manuscript with Track Changes'.
- An unmarked version of your revised paper without tracked changes. You should upload this as a separate file labeled 'Manuscript'.

If you would like to make changes to your financial disclosure, please include your updated statement in your cover letter. Guidelines for resubmitting your figure files are available below the reviewer comments at the end of this letter.

If applicable, we recommend that you deposit your laboratory protocols in protocols.io to enhance the reproducibility of your results. Protocols.io assigns your protocol its own identifier (DOI) so that it can be cited independently in the future. For instructions see: https://journals.plos.org/plosone/s/submission-guidelines#loc-laboratory-protocols. Additionally, PLOS ONE offers an option for publishing peer-reviewed Lab Protocol articles, which describe protocols hosted on protocols.io. Read more information on sharing protocols at https://plos.org/protocols?utm_medium=editorial-email&utm_source=authorletters&utm_campaign=protocols.

As the corresponding author, your ORCID iD is verified in the submission system and will appear in the published article. PLOS supports the use of ORCID, and we encourage all coauthors to register for an ORCID iD and use it as well. Please encourage your coauthors to verify their ORCID iD within the submission system before final acceptance, as unverified ORCID iDs will not appear in the published article. *Only*  the individual author can complete the verification step; PLOS staff *cannot*  verify ORCID iDs on behalf of authors.

We look forward to receiving your revised manuscript.

Kind regards,

Philip A. Cola, Ph.D.

Academic Editor

PLOS One

Journal Requirements:

1. If the reviewer comments include a recommendation to cite specific previously published works, please review and evaluate these publications to determine whether they are relevant and should be cited. There is no requirement to cite these works unless the editor has indicated otherwise.

2. Please review your reference list to ensure that it is complete and correct. If you have cited papers that have been retracted, please include the rationale for doing so in the manuscript text, or remove these references and replace them with relevant current references. Any changes to the reference list should be mentioned in the rebuttal letter that accompanies your revised manuscript. If you need to cite a retracted article, indicate the article’s retracted status in the References list and also include a citation and full reference for the retraction notice.

Additional Editor Comments:

We appreciated the revised version of your manuscript. The reviewers and I agree that the paper has improved substantially since the previous round of review. In particular, the revised manuscript presents a clearer conceptual framing, stronger methodological justification, improved thematic organization, a more nuanced discussion of health-data governance, and more actionable implications for interdisciplinary AI-healthcare research. The revisions have significantly strengthened the manuscript’s contribution and overall readability.

At the same time, several minor issues remain before the manuscript can be considered for publication. The principal area requiring attention is ensuring that claims regarding patient and public involvement accurately reflect the study’s interview sample and evidentiary base. In addition, the reviewers recommend several modest clarifications related to methodological transparency, sampling-frame description, data availability, and consistency of terminology and presentation throughout the manuscript. These requested revisions are limited in scope and do not require additional data collection or substantial reanalysis. Accordingly, I am inviting you to submit a revised manuscript that addresses the reviewers’ comments that you will see below.

I would like to thank the authors for their thoughtful and comprehensive response to the previous round of reviews as the manuscript has been improved. Both of the current reviewers note that the manuscript has been substantially strengthened through revision. In particular, the introduction now provides a clearer and more focused rationale for the study, the methodological transparency has improved considerably, the sample-size justification using information power is well articulated, and the findings and discussion are more coherently organized and accessible. Reviewers also highlighted improvements in the treatment of health-data governance, the reframing of the “AI knowledge sharing” theme, and the addition of more practical implications for interdisciplinary AI-healthcare collaborations. Collectively, these revisions have significantly enhanced the manuscript’s clarity, rigor, and contribution to understanding collaborative practices in the early stages of AI development for healthcare.

While the manuscript is now close to publication readiness, the reviewers identified several relatively minor issues that should be addressed in a further revision. The most important concern is ensuring that claims regarding patient and public involvement accurately reflect the evidence produced by the conduct of the study. Because the interview sample consisted of consortium members, including PPIE coordinators, rather than patients or public contributors themselves, references throughout the abstract, findings, discussion, and conclusion should consistently distinguish between direct patient perspectives and participants’ accounts of patient/public involvement. In addition, the reviewers recommend several modest enhancements to methodological transparency, including clarification regarding member checking, the rationale for not using quantitative inter-coder reliability measures, the demographic information collected, and the development and inclusion of the interview guide. Further clarification of the sampling frame, inclusion of a clear data availability statement, resolution of several minor issues of wording and conceptual framing, and a careful final copyedit for typographical errors and terminology consistency would also strengthen the manuscript. These revisions are limited in scope and do not require additional data collection or substantive reanalysis. Accordingly, I am recommending revise and resubmit (minor revisions) and encourage the authors to address each reviewer comment carefully in the next version.

[Note: HTML markup is below. Please do not edit.]

Reviewer's Responses to Questions

**Comments to the Author**

1. If the authors have adequately addressed your comments raised in a previous round of review and you feel that this manuscript is now acceptable for publication, you may indicate that here to bypass the “Comments to the Author” section, enter your conflict of interest statement in the “Confidential to Editor” section, and submit your "Accept" recommendation.

Reviewer #3: All comments have been addressed

Reviewer #4: (No Response)

**********

2. Is the manuscript technically sound, and do the data support the conclusions?

The manuscript must describe a technically sound piece of scientific research with data that supports the conclusions. Experiments must have been conducted rigorously, with appropriate controls, replication, and sample sizes. The conclusions must be drawn appropriately based on the data presented.

Reviewer #3: Yes

Reviewer #4: Partly

**********

3. Has the statistical analysis been performed appropriately and rigorously?

Reviewer #3: N/A

Reviewer #4: N/A

**********

4. Have the authors made all data underlying the findings in their manuscript fully available?

The PLOS Data policy requires authors to make all data underlying the findings described in their manuscript fully available without restriction, with rare exception (please refer to the Data Availability Statement in the manuscript PDF file). The data should be provided as part of the manuscript or its supporting information, or deposited to a public repository. For example, in addition to summary statistics, the data points behind means, medians and variance measures should be available. If there are restrictions on publicly sharing data—e.g. participant privacy or use of data from a third party—those must be specified.

Reviewer #3: Yes

Reviewer #4: No

**********

5. Is the manuscript presented in an intelligible fashion and written in standard English?

PLOS ONE does not copyedit accepted manuscripts, so the language in submitted articles must be clear, correct, and unambiguous. Any typographical or grammatical errors should be corrected at revision, so please note any specific errors here.

Reviewer #3: Yes

Reviewer #4: Yes

**********

6. Review Comments to the Author

Please use the space provided to explain your answers to the questions above. You may also include additional comments for the author, including concerns about dual publication, research ethics, or publication ethics. (Please upload your review as an attachment if it exceeds 20,000 characters)

Reviewer #3: This manuscript presents a qualitative analysis of collaborative AI research in healthcare contexts during formative stages (pre-bedside). The impetus of this work is that newer AI technologies present new opportunities for application in healthcare, and there is little know about challenges and solutions to manage collaboration, which is key to developing translatable AI-powered solutions for patient care and healthcare systems. In the revised manuscript, prior reviewer comments largely addressed. In particular, the streamlined introduction has significantly improved the organization and readability of the paper, as well as the inclusion of information on disciplinary background of participants. The sample size justification is particularly strong. My comments are minor and intended to further strengthen clarity and presentation.

1) At the end of the introduction, the manuscript refers to PPIE Coordinators (second bullet, page 6) before these roles are clearly introduced. While it is appropriate that detailed information about the consortia was moved to the Methods section, it may be helpful to retain a brief description in the introduction, including a note about the role of the PPIE coordinators. Alternatively, the authors could remove this reference in the introduction and slightly broaden the stated study purpose. Either approach would improve clarity and flow.

2) In the opening of the Findings section (page 16, second sentence), the statement describing how AI research “impacts those that are not familiar with it” may be overly broad. Consider narrowing this language to better align with the data presented. For example, the statement could focus on participation in AI research or emphasize participants’ perceptions of AI’s impact, either of which may more accurately reflect the findings.

3) The term National Safe Havens is inconsistently capitalized throughout the manuscript. Please standardize its usage for consistency.

4) While there are different approaches to presenting qualitative findings, one interpretive statement at the end of the first theme (page 24) appears relatively strong for the Findings section. Specifically, the assertion that AI development is a socio technical endeavor influenced by technical factors seems to reflect an a priori assumption stated earlier in the manuscript. I recommend moving this statement to the Discussion, where it could effectively reinforce how the study’s findings align with the conceptual framework guiding the research.

Reviewer #4: Thank you for the opportunity to review the revised manuscript, “‘We’ll have to see how it works’: An interview study to understand collaborative practices in interdisciplinary artificial intelligence and healthcare research.” As a matter of record, I declare no conflicts of interest in reviewing this submission.

I was asked to evaluate the authors’ revised submission given the feedback provided during a prior round of peer review. Accordingly, I have focused primarily on whether the authors have sufficiently addressed the earlier reviewers’ comments, while also noting a few residual issues that remain relevant as they relate to PLOS One’s publication criteria. I found the manuscript substantially improved from its original submission. The authors have made revisions that materially strengthen the conceptual framing, methodological transparency, and presentation of findings.

The authors examine how AI work practices complicate interdisciplinary work in healthcare research, drawing from semi-structured interviews with 13 participants across three UK-based research consortia focused on AI and multimorbidity. Through inductive thematic analysis, they identify three areas of collaborative practice: AI novelty, AI knowledge sharing, and the inclusion of multiple real-world healthcare perspectives. The authors position the study within a sociotechnical theory of AI, where technical choices about models, data, tools, and computational infrastructure are inseparable from the social, institutional, and disciplinary arrangements through which AI development occurs.

This is an interesting and worthwhile setting. Literature around AI in healthcare tends to focus either on the implementation of AI systems in clinical settings or on technical/model performance considerations. As such, the attention given here to the formative stages of AI development prior to deployment is useful. The study’s strongest contribution is that it offers a focused glimpse into how members of AI-healthcare research consortia describe the iterative and often underappreciated work of making AI development understandable across disciplinary boundaries.

The authors have made several meaningful improvements. The revised Introduction is much clearer than the original submission. The original Related Work section has been condensed into a more coherent introductory argument, and the authors now more directly explain why AI specifically complicates interdisciplinary collaboration. The revised manuscript gives greater attention to computational complexity, large dataset requirements, the black-box character of some AI methods, restrictions associated with health data, and the need for various stakeholder perspectives to help interpret or contextualize model development. This is a strong improvement because the paper now better explains why AI is not simply another health technology, but a technology whose development amplifies existing collaborative tensions.

The authors have also improved the methods section. The addition of Malterud et al.’s information power rationale is appropriate for such a qualitative study, and a basic clarification of eligibility criteria is helpful. The authors also added interviewer background, described the coding process with greater clarity, and submitted an SRQR checklist. Likewise, the findings are more accessible now that “Communicating AI” has been renamed and reframed as “AI knowledge sharing.” This resolves an important ambiguity from the earlier manuscript, because the prior label could be read as communication with AI rather than communication about AI-related work. The revised theme is more precise and better linked to the collaborative practices described in the interviews.

The discussion of health data access has also improved considerably. The original wording risked treating data governance, privacy, and access restrictions solely as frustrating barriers. In the revised manuscript, the authors more carefully acknowledge that health records are personal data subject to ethical approvals, anonymization requirements, safe havens, output checking, and disclosure-risk constraints. This revision is important because it preserves the authors’ empirical point, that data access and safe-haven infrastructures shape collaborative AI work, without diminishing the ethical reasons such safeguards exist. Finally, the revised Conclusion now includes more pragmatic suggestions about expectation-setting, AI literacy, support for PPIE coordinators, and resourcing the work of interdisciplinary coordination. These additions are appropriate and help translate the findings into actionable implications.

Notwithstanding these improvements, I believe several minor revisions remain necessary before the manuscript is ready for publication. These are not issues that require new data collection or a major reanalysis. I outline each of these issues below.

(1) Claim Discipline

The first and most important remaining concern is that the manuscript still sometimes overstates the evidentiary basis for claims about patients, patient representatives, and PPIE participants. The limitation section appropriately acknowledges that no patients from the PPIE groups were interviewed. This is an important clarification, and I appreciate that the authors have included it. However, this limitation is not yet carried consistently through the abstract, findings, discussion, and conclusion. In several places, the manuscript still reads as though direct patient or patient-representative perspectives were captured, when the direct interview evidence appears to come from consortium members, including PPIE coordinators, rather than from patients or lay contributors themselves. I have articulated some examples of where this remains an issue in the line-by-line feedback that follows these general comments.

Despite this concern, it is not a fatal issue for the manuscript. Indeed, one of the manuscript’s more interesting findings is that PPIE coordinators appear to take on a translational or knowledge-brokering role by adapting AI-related materials for patient/public audiences and helping researchers understand how such materials may be received. That is an important contribution. However, the authors should more consistently distinguish between direct interview evidence from PPIE coordinators and indirect accounts of patient/public involvement. In many places, the manuscript could be strengthened by using language such as “PPIE coordinators’ accounts of patient/public involvement,” “participants’ descriptions of PPIE engagement,” or “reported patient/public contributions,” rather than phrasing the findings as though patient perspectives were directly elicited.

A related issue concerns the theme of “multiple real-world healthcare perspectives.” I understand what the authors are trying to accomplish with this theme. Clinicians and patients/public contributors can help contextualize electronic health record data, interpret findings, identify practical relevance, and remind technical teams that data corresponds to real patient experiences. However, in the present study, the patient/public dimension is mediated through PPIE coordinators and consortium participants’ accounts. The theme can remain, but the evidentiary basis should be stated more carefully. Otherwise, the paper risks overselling its sample, which was one of the central concerns raised by the initial reviewers.

(2) Methodological Transparency

The second remaining issue concerns methodological transparency. The revised methods section is improved, and the SRQR checklist confirms that the authors have attempted to map the manuscript against recognized qualitative reporting standards. Still, several details are clearer in the authors’ response to the initial reviewers than in the manuscript itself. I recommend adding a short paragraph to the methods or limitations section that explicitly addresses four points.

First, the authors should state whether participant validation or member checking was conducted. The response letter indicates that participants were not shown results or contacted after coding. However, this has not been translated into the revised manuscript. This does not invalidate the analysis, but it should be disclosed plainly in the manuscript.

Second, the authors should briefly explain why a quantitative inter-coder agreement statistic, such as kappa, was not used. I agree that kappa is not necessarily appropriate for Braun and Clarke-style thematic analysis, particularly when the analysis is inductive and interpretive rather than grounded in a strictly positivist coding ontology. However, the rationale should appear in the manuscript rather than only in the response letter. The authors can point readers to the procedures they did use to enhance analytic trustworthiness, such as initial independent coding, harmonization of code vocabulary, codebook refinement, revisiting early transcripts, discussion of coding changes, and joint development of themes.

Third, the authors should clarify what demographic information was and was not collected. The response letter indicates that age, sex, and gender were not collected because the authors viewed role, expertise, and AI experience as more relevant to the study aims. That may be reasonable, particularly in a small and potentially identifiable sample. However, it should be stated transparently in the manuscript. The authors might also frame this as a balance between contextualizing participants and minimizing deductive disclosure risk in a small sample.

Fourth, the interview guide is summarized, but not provided. I do not think the absence of the full interview guide prevents publication, but the manuscript would be strengthened either by including it as supporting information or by giving a somewhat fuller account of how the guide was developed from the literature, prior questions, domain knowledge, informal consultation, and piloting. This would directly respond to the prior reviewer’s concern about whether a validated framework guided the interview protocol.

(3) Sampling-Frame Precision

The third issue is sampling-frame precision. The addition of Malterud et al.’s information power rationale is appropriate and helps justify the sample size. However, the eligible population remains somewhat unclear. The revised manuscript states that each consortium employs around 30 academic and healthcare researchers, while the authors’ response letter refers to around 20 research staff per consortium and frames the sample as 13 participants from approximately 60 potential participants. This may simply reflect different definitions of “research staff,” “formal consortium members,” and broader consortium contributors. However, because the sample-size justification depends partly on the size of the eligible pool, the manuscript should clarify the denominator. If approximately 30 people per consortium includes all academic, clinical, and affiliated contributors, but approximately 20 per consortium refers only to formal research staff practically eligible or reachable for interviews, the authors should state that distinction.

(4) Data Availability

The fourth issue concerns data availability. I did not see a clear manuscript-level data availability statement in the version I reviewed. I recognize that qualitative interview transcripts from a small number of identifiable research consortia may not be appropriate for full public sharing, even if deidentified, because of disclosure risk. That restriction may be entirely justified. However, the manuscript should clearly explain what data can and cannot be shared, and why. At minimum, the authors should specify whether full transcripts are unavailable for confidentiality reasons, whether deidentified excerpts can be made available, and whether the interview guide or codebook can be shared as supporting information.

(5) Final Copyediting Considerations

Finally, the manuscript is intelligible and generally written in standard English, but it still needs careful copyediting before publication. The remaining errors do not undermine the study, but they are distracting and should be corrected. PLOS One does not copyedit accepted manuscripts, and I encourage the authors to give the paper a final close review for typographical errors, table formatting, and consistency of terminology.

Specific highlights and points of suggested improvement are listed below:

(Lines 14-28, Abstract) – The abstract is much improved in its framing of the study, but it still overstates patient involvement. The phrase indicating that AI development brings together “data scientists, clinicians, patients and other stakeholders” is broadly true of the consortia, but not of the interview sample. Likewise, the statement that the study shows how “clinicians and patient representatives are involved” should be revised to reflect the actual evidence. A more accurate formulation might refer to how consortium participants, including clinicians and PPIE coordinators, describe the involvement of clinical and patient/public perspectives in early-stage AI development.

(Lines 91-104, Research Question) – The research question remains somewhat awkwardly worded: “how do AI work practices trouble ways of interdisciplinary work in healthcare research?” The meaning is understandable, but the phrasing is not yet polished. Consider revising to something like: “How do AI work practices complicate interdisciplinary collaboration in healthcare research?” or “How do AI work practices shape interdisciplinary collaboration in early-stage healthcare research?”

(Lines 106-118, Contributions) – The second contribution statement is much clearer than in the earlier manuscript, but it should be careful not to overstate patient evidence. The reference to PPIE coordinators bridging communication between researchers and patients is appropriate. Claims about “ensur[ing] these voices are heard” should be phrased so the reader understands that this conclusion is based on interviews with consortium participants and PPIE coordinators, not direct interviews with patients/public contributors.

(Lines 122-143, Consortia at a glance) – This section is appropriately located in the methods and gives useful context. However, the statement that each consortium employs around 30 academic and healthcare researchers should be reconciled with the response-letter statement that each consortium had around 20 research staff. This matters because it is later used, indirectly, to justify sample size.

(Lines 146-166, Participants) – The use of Malterud et al.’s information-power framework is appropriate. However, the eligibility sentence requires correction and clarification. The phrase “reachable my email” appears to be a typographical error. Beyond the typo, the authors should clarify whether “any member of the consortia” includes external PPIE contributors or only formal consortium members. This clarification is especially important because the absence of direct patient/public interviews is a recurring limitation.

(Table 1, Lines 168-169) – The addition of “Data science” to the two postdoctoral research associates is helpful. The table formatting could be improved, as the current version is difficult to read. As a starting point, consider widening the table dimensions to the maximum possible width.

(Lines 172-177, Interviews) – The authors now identify the background of RH and ER, which is helpful. The development of the interview guide remains somewhat brief. Consider adding one or two sentences describing how the literature, domain knowledge, informal consultation, and pilot interview shaped the final guide. Alternatively (or additionally), the interview guide could be provided as supporting information.

(Lines 190-205, Creation of codebook and analysis) – The coding process is reasonably described. However, “Braun and Clark” should be corrected to “Braun and Clarke.” This section would also benefit from a brief explanation of trustworthiness procedures and the rationale for not reporting kappa. I would not require kappa for this kind of thematic analysis, but the manuscript should explain why interpretive thematic analysis was better served by iterative discussion, codebook refinement, and joint theme development rather than a quantitative inter-coder reliability statistic.

(Lines 208-214, Findings introduction) – The penultimate sentence of this first paragraph again describes the third theme as involving “clinicians and patients.” Since no patients were interviewed, this should be revised. “Clinicians and PPIE coordinators’ accounts of patient/public involvement” would be more accurate.

(Lines 342-444, AI knowledge sharing) – The reframing from “Communicating AI” to “AI knowledge sharing” is a strong revision. This section now more clearly captures how participants adapted tools, formats, and levels of technical detail for different audiences. Some minor grammatical issues remain, but the analytic structure is clearer.

(Lines 484-530, Relevance to patients) – This is an important part of the findings, but the authors should revise the language to make clear when claims are based on PPIE coordinator accounts rather than direct patient interview evidence. Phrases such as “Patients particularly emphasised” and “Patient collaborators help” should be checked against the available interview data. If these claims are based on what PPIE coordinators reported, that should be stated.

(Lines 624-662, Additional perspectives in collaborative AI research) – This section contains some of the manuscript’s most interesting discussion, especially the role of PPIE coordinators as translators or knowledge brokers. However, the language again needs careful calibration. The study does not directly show how patients influence AI work practices. It shows how consortium participants, including PPIE coordinators, describe patient/public involvement and its mediation through consortium practices.

(Lines 669-695, Limitations and future work) – The limitations section appropriately acknowledges the UK/NHS context, early-stage consortia, and the absence of direct patient interviews. This section is helpful. However, these limitations should be reflected more consistently in the abstract, findings, discussion, and conclusion rather than appearing only toward the end of the paper.

(Line-level copyediting) – Please correct remaining typographical and grammatical issues, including “reachable my email,” “Braun and Clark,” “Juypter notebook,” and phrases such as “data scientists needed to adjust their adapting communication styles.” Please also check consistency in the use of “multidisciplinary” versus “interdisciplinary,” “PPIE participants” versus “PPIE contributors,” “patient representatives” versus “patients,” and “PPIE coordinators.”

In summary, the revised manuscript is substantially stronger than the original submission. The authors have clarified the conceptual framing, improved the organization of the manuscript, provided a more defensible sample-size rationale, refined the thematic structure, improved the discussion of health-data governance, and added useful practical recommendations. The remaining revisions are focused and manageable. With these minor revisions, the manuscript would be much better positioned for publication.

**********

7. PLOS authors have the option to publish the peer review history of their article (what does this mean?). If published, this will include your full peer review and any attached files.

If you choose “no”, your identity will remain anonymous but your review may still be made public.

**Do you want your identity to be public for this peer review?**  For information about this choice, including consent withdrawal, please see our Privacy Policy.

Reviewer #3: No

Reviewer #4: **Yes:** Joshua A. Gerlick

**********

[NOTE: If reviewer comments were submitted as an attachment file, they will be attached to this email and accessible via the submission site. Please log into your account, locate the manuscript record, and check for the action link "View Attachments". If this link does not appear, there are no attachment files.]

To ensure your figures meet our technical requirements, please review our figure guidelines: https://journals.plos.org/plosone/s/figures

You may also use PLOS’s free figure tool, NAAS, to help you prepare publication quality figures: https://journals.plos.org/plosone/s/figures#loc-tools-for-figure-preparation.

NAAS will assess whether your figures meet our technical requirements by comparing each figure against our figure specifications.

# Author comment: Author response to Decision Letter 2

**DOI:** 10.1371/journal.pone.0354358.r004

19 Jun 2026

Dear editor and reviewers,

We are thankful for the invitation to submit a minor revision to our manuscript. We edited the manuscript in response to the feedback provided and addressed the reviewers' points. We clarified the involvement of PPIE coordinators rather than patients or members of the public throughout the manuscript and added additional methodological explanations as requested. In the Response to Reviewers we address each point as requested and signpost to the pages and lines on the paper where they can be found.

Best regards,

Rafael Henkin,

on behalf of the authors

Response to Reviewers

--

Review Comments to the Author

Please use the space provided to explain your answers to the questions above. You may also include additional comments for the author, including concerns about dual publication, research ethics, or publication ethics. (Please upload your review as an attachment if it exceeds 20,000 characters)

Reviewer #3: This manuscript presents a qualitative analysis of collaborative AI research in healthcare contexts during formative stages (pre-bedside). The impetus of this work is that newer AI technologies present new opportunities for application in healthcare, and there is little know about challenges and solutions to manage collaboration, which is key to developing translatable AI-powered solutions for patient care and healthcare systems. In the revised manuscript, prior reviewer comments largely addressed. In particular, the streamlined introduction has significantly improved the organization and readability of the paper, as well as the inclusion of information on disciplinary background of participants. The sample size justification is particularly strong. My comments are minor and intended to further strengthen clarity and presentation.

1) At the end of the introduction, the manuscript refers to PPIE Coordinators (second bullet, page 6) before these roles are clearly introduced. While it is appropriate that detailed information about the consortia was moved to the Methods section, it may be helpful to retain a brief description in the introduction, including a note about the role of the PPIE coordinators. Alternatively, the authors could remove this reference in the introduction and slightly broaden the stated study purpose. Either approach would improve clarity and flow.

We have updated the second bullet point to reflect that this is staff engaged in PPIE work. This extends further than just PPIE coordinators, as most researchers also engaged in PPIE work and changed practices to work with PPIE contributors. We expand and define the role of PPIE coordinators in the Methods section.

2) In the opening of the Findings section (page 16, second sentence), the statement describing how AI research “impacts those that are not familiar with it” may be overly broad. Consider narrowing this language to better align with the data presented. For example, the statement could focus on participation in AI research or emphasize participants’ perceptions of AI’s impact, either of which may more accurately reflect the findings.

Thanks, we have updated the opening (page 10 in clean version) to reflect the main results more closely and have replaced the line “impacts those that are not familiar with it” with “We discuss how participation in AI research is often novel for participants resulting in different expectations of the capabilities of AI and how this novelty can also amplify the differences between disciplines as stakeholders learn new ways of working.”

3) The term National Safe Havens is inconsistently capitalized throughout the manuscript. Please standardize its usage for consistency.

Thank you, we have ensured consistency throughout.

4) While there are different approaches to presenting qualitative findings, one interpretive statement at the end of the first theme (page 24) appears relatively strong for the Findings section. Specifically, the assertion that AI development is a socio technical endeavor influenced by technical factors seems to reflect an a priori assumption stated earlier in the manuscript. I recommend moving this statement to the Discussion, where it could effectively reinforce how the study’s findings align with the conceptual framework guiding the research.

We have moved the socio-technical sentence to the beginning of the discussion (page 24 in clean version).

Reviewer #4: Thank you for the opportunity to review the revised manuscript, “‘We’ll have to see how it works’: An interview study to understand collaborative practices in interdisciplinary artificial intelligence and healthcare research.” As a matter of record, I declare no conflicts of interest in reviewing this submission.

I was asked to evaluate the authors’ revised submission given the feedback provided during a prior round of peer review. Accordingly, I have focused primarily on whether the authors have sufficiently addressed the earlier reviewers’ comments, while also noting a few residual issues that remain relevant as they relate to PLOS One’s publication criteria. I found the manuscript substantially improved from its original submission. The authors have made revisions that materially strengthen the conceptual framing, methodological transparency, and presentation of findings.

The authors examine how AI work practices complicate interdisciplinary work in healthcare research, drawing from semi-structured interviews with 13 participants across three UK-based research consortia focused on AI and multimorbidity. Through inductive thematic analysis, they identify three areas of collaborative practice: AI novelty, AI knowledge sharing, and the inclusion of multiple real-world healthcare perspectives. The authors position the study within a sociotechnical theory of AI, where technical choices about models, data, tools, and computational infrastructure are inseparable from the social, institutional, and disciplinary arrangements through which AI development occurs.

This is an interesting and worthwhile setting. Literature around AI in healthcare tends to focus either on the implementation of AI systems in clinical settings or on technical/model performance considerations. As such, the attention given here to the formative stages of AI development prior to deployment is useful. The study’s strongest contribution is that it offers a focused glimpse into how members of AI-healthcare research consortia describe the iterative and often underappreciated work of making AI development understandable across disciplinary boundaries.

The authors have made several meaningful improvements. The revised Introduction is much clearer than the original submission. The original Related Work section has been condensed into a more coherent introductory argument, and the authors now more directly explain why AI specifically complicates interdisciplinary collaboration. The revised manuscript gives greater attention to computational complexity, large dataset requirements, the black-box character of some AI methods, restrictions associated with health data, and the need for various stakeholder perspectives to help interpret or contextualize model development. This is a strong improvement because the paper now better explains why AI is not simply another health technology, but a technology whose development amplifies existing collaborative tensions.

The authors have also improved the methods section. The addition of Malterud et al.’s information power rationale is appropriate for such a qualitative study, and a basic clarification of eligibility criteria is helpful. The authors also added interviewer background, described the coding process with greater clarity, and submitted an SRQR checklist. Likewise, the findings are more accessible now that “Communicating AI” has been renamed and reframed as “AI knowledge sharing.” This resolves an important ambiguity from the earlier manuscript, because the prior label could be read as communication with AI rather than communication about AI-related work. The revised theme is more precise and better linked to the collaborative practices described in the interviews.

The discussion of health data access has also improved considerably. The original wording risked treating data governance, privacy, and access restrictions solely as frustrating barriers. In the revised manuscript, the authors more carefully acknowledge that health records are personal data subject to ethical approvals, anonymization requirements, safe havens, output checking, and disclosure-risk constraints. This revision is important because it preserves the authors’ empirical point, that data access and safe-haven infrastructures shape collaborative AI work, without diminishing the ethical reasons such safeguards exist. Finally, the revised Conclusion now includes more pragmatic suggestions about expectation-setting, AI literacy, support for PPIE coordinators, and resourcing the work of interdisciplinary coordination. These additions are appropriate and help translate the findings into actionable implications.

Notwithstanding these improvements, I believe several minor revisions remain necessary before the manuscript is ready for publication. These are not issues that require new data collection or a major reanalysis. I outline each of these issues below.

(1) Claim Discipline

The first and most important remaining concern is that the manuscript still sometimes overstates the evidentiary basis for claims about patients, patient representatives, and PPIE participants. The limitation section appropriately acknowledges that no patients from the PPIE groups were interviewed. This is an important clarification, and I appreciate that the authors have included it. However, this limitation is not yet carried consistently through the abstract, findings, discussion, and conclusion. In several places, the manuscript still reads as though direct patient or patient-representative perspectives were captured, when the direct interview evidence appears to come from consortium members, including PPIE coordinators, rather than from patients or lay contributors themselves. I have articulated some examples of where this remains an issue in the line-by-line feedback that follows these general comments.

Despite this concern, it is not a fatal issue for the manuscript. Indeed, one of the manuscript’s more interesting findings is that PPIE coordinators appear to take on a translational or knowledge-brokering role by adapting AI-related materials for patient/public audiences and helping researchers understand how such materials may be received. That is an important contribution. However, the authors should more consistently distinguish between direct interview evidence from PPIE coordinators and indirect accounts of patient/public involvement. In many places, the manuscript could be strengthened by using language such as “PPIE coordinators’ accounts of patient/public involvement,” “participants’ descriptions of PPIE engagement,” or “reported patient/public contributions,” rather than phrasing the findings as though patient perspectives were directly elicited.

A related issue concerns the theme of “multiple real-world healthcare perspectives.” I understand what the authors are trying to accomplish with this theme. Clinicians and patients/public contributors can help contextualize electronic health record data, interpret findings, identify practical relevance, and remind technical teams that data corresponds to real patient experiences. However, in the present study, the patient/public dimension is mediated through PPIE coordinators and consortium participants’ accounts. The theme can remain, but the evidentiary basis should be stated more carefully. Otherwise, the paper risks overselling its sample, which was one of the central concerns raised by the initial reviewers.

Thank you, we have added additional clarity and changed the wording both in the findings (page 10 in the clean version) and discussion (page 24) to highlight that these are reported patient contributions, rather than directly evidenced by patients as interviewees.

(2) Methodological Transparency

The second remaining issue concerns methodological transparency. The revised methods section is improved, and the SRQR checklist confirms that the authors have attempted to map the manuscript against recognized qualitative reporting standards. Still, several details are clearer in the authors’ response to the initial reviewers than in the manuscript itself. I recommend adding a short paragraph to the methods or limitations section that explicitly addresses four points.

First, the authors should state whether participant validation or member checking was conducted. The response letter indicates that participants were not shown results or contacted after coding. However, this has not been translated into the revised manuscript. This does not invalidate the analysis, but it should be disclosed plainly in the manuscript.

Thank you, we have added a line on page 9 to state that participants were active in the interviews only and were not involved in the coding or discussion of the results.

Second, the authors should briefly explain why a quantitative inter-coder agreement statistic, such as kappa, was not used. I agree that kappa is not necessarily appropriate for Braun and Clarke-style thematic analysis, particularly when the analysis is inductive and interpretive rather than grounded in a strictly positivist coding ontology. However, the rationale should appear in the manuscript rather than only in the response letter. The authors can point readers to the procedures they did use to enhance analytic trustworthiness, such as initial independent coding, harmonization of code vocabulary, codebook refinement, revisiting early transcripts, discussion of coding changes, and joint development of themes.

We have addressed this comment by including on page 10 a brief rationale related to analytic rigour and the Braun and Clarke methodology, as to why Cohen’s kappa or other statistics were not utilised.

Third, the authors should clarify what demographic information was and was not collected. The response letter indicates that age, sex, and gender were not collected because the authors viewed role, expertise, and AI experience as more relevant to the study aims. That may be reasonable, particularly in a small and potentially identifiable sample. However, it should be stated transparently in the manuscript. The authors might also frame this as a balance between contextualizing participants and minimizing deductive disclosure risk in a small sample.

We thank the reviewer for highlighting this. On page 9, we have included a sentence on the data collected and why demographic data was not included.

Fourth, the interview guide is summarized, but not provided. I do not think the absence of the full interview guide prevents publication, but the manuscript would be strengthened either by including it as supporting information or by giving a somewhat fuller account of how the guide was developed from the literature, prior questions, domain knowledge, informal consultation, and piloting. This would directly respond to the prior reviewer’s concern about whether a validated framework guided the interview protocol.

We have added additional details on page 8 ofhow the interview guide was developed and the questions it contained which were based on readings from the literature, as well as informed by the pilot study and feedback from the interdisciplinary research team.

(3) Sampling-Frame Precision

The third issue is sampling-frame precision. The addition of Malterud et al.’s information power rationale is appropriate and helps justify the sample size. However, the eligible population remains somewhat unclear. The revised manuscript states that each consortium employs around 30 academic and healthcare researchers, while the authors’ response letter refers to around 20 research staff per consortium and frames the sample as 13 participants from approximately 60 potential participants. This may simply reflect different definitions of “research staff,” “formal consortium members,” and broader consortium contributors. However, because the sample-size justification depends partly on the size of the elig

Attachment Submitted filename: minor_revision_comments.docx

# Editor report: Decision Letter 2

**DOI:** 10.1371/journal.pone.0354358.r005

**Authors:** Philip A. Cola

8 Jul 2026

“We’ll have to see how it works”: An interview study to understand collaborative practices in interdisciplinary artificial intelligence and healthcare research

PONE-D-25-21991R2

Dear Dr. Henkin,

We’re pleased to inform you that your manuscript has been judged scientifically suitable for publication and will be formally accepted for publication once it meets all outstanding technical requirements.

Within one week, you’ll receive an e-mail detailing the required amendments. When these have been addressed, you’ll receive a formal acceptance letter and your manuscript will be scheduled for publication.

An invoice will be generated when your article is formally accepted. Please note, if your institution has a publishing partnership with PLOS and your article meets the relevant criteria, all or part of your publication costs will be covered. Please make sure your user information is up-to-date by logging into Editorial Manager at Editorial Manager® and clicking the ‘Update My Information' link at the top of the page. For questions related to billing, please contact billing support.

If your institution or institutions have a press office, please notify them about your upcoming paper to help maximize its impact. If they’ll be preparing press materials, please inform our press team as soon as possible -- no later than 48 hours after receiving the formal acceptance. Your manuscript will remain under strict press embargo until 2 pm Eastern Time on the date of publication. For more information, please contact onepress@plos.org.

Kind regards,

Philip A. Cola, Ph.D.

Academic Editor

PLOS One

Additional Editor Comments (optional):

Thank you for your careful revision and detailed response to both sets of comments from the reviewers. The manuscript has been substantially strengthened through improved conceptual framing, greater methodological transparency, clearer presentation of the findings, and more appropriate calibration of the study's claims. The reviewers' substantive concerns even thought both requested minor revisions have been satisfactorily addressed, and the remaining issues are likely editorial in nature. I am pleased to recommend acceptance for publication, subject to final editorial and production checks to ensure consistency of terminology as it appears that you have incorporated the revisions described in the response letter accordingly and based on my review.

# Editor report: Acceptance letter

**DOI:** 10.1371/journal.pone.0354358.r006

**Authors:** Philip A. Cola

PONE-D-25-21991R2

PLOS One

Dear Dr. Henkin,

I'm pleased to inform you that your manuscript has been deemed suitable for publication in PLOS One. Congratulations! Your manuscript is now being handed over to our production team.

At this stage, our production department will prepare your paper for publication. This includes ensuring the following:

* All references, tables, and figures are properly cited

* All relevant supporting information is included in the manuscript submission,

* There are no issues that prevent the paper from being properly typeset

You will receive further instructions from the production team, including instructions on how to review your proof when it is ready. Please keep in mind that we are working through a large volume of accepted articles, so please give us a few days to review your paper and let you know the next and final steps.

Lastly, if your institution or institutions have a press office, please let them know about your upcoming paper now to help maximize its impact. If they'll be preparing press materials, please inform our press team within the next 48 hours. Your manuscript will remain under strict press embargo until 2 pm Eastern Time on the date of publication. For more information, please contact onepress@plos.org.

You will receive an invoice from PLOS for your publication fee after your manuscript has reached the completed accept phase. If you receive an email requesting payment before acceptance or for any other service, this may be a phishing scheme. Learn how to identify phishing emails and protect your accounts at https://explore.plos.org/phishing.

If we can help with anything else, please email us at customercare@plos.org.

Thank you for submitting your work to PLOS ONE and supporting open access.

Kind regards,

PLOS ONE Editorial Office Staff

on behalf of

Dr. Philip A. Cola

Academic Editor

PLOS One
